# Exposure to heavy metals and trace elements and risk of dyslipidemia: a nested case-control analysis in rural adults

**DOI:** 10.3389/fendo.2026.1851531

**Published:** 2026-05-21

**Authors:** Jiangwei Qiu, Qingan Wang, Jiaxing Zhang, Chen Chen, Kexin Chen, Yuhong Zhang, Yi Zhao

**Affiliations:** 1 School of Public Health, Ningxia Medical University, Yinchuan, China; 2 Key Laboratory of Environmental Factors and Chronic Disease Control, Ningxia Medical University, Yinchuan, China; 3 Department of Public Health, People’s Hospital of Ningxia Hui Autonomous Region, Ningxia Medical University, Yinchuan, China; 4 School of Nursing, Ningxia Medical University, Yinchuan, China

**Keywords:** dyslipidemia, heavy metals, nested case-control study, rural population, trace elements

## Abstract

**Background:**

Exposure to some conventional metal trace elements has been found to be associated with abnormal blood lipids; however, evidence for combined exposure is inconclusive. This study aimed to explore the joint associations between multiple metal trace elements and dyslipidemia.

**Methods:**

In this nested case-control study, 782 subjects (391 cases and 391 controls, 1:1 case-to-control ratio) were selected from a cohort in Ningxia, China. Levels of 15 trace metals in serum were determined by inductively coupled plasma atomic emission spectrometry. Conditional logistic regression was used to analyze association of dyslipidemia. Bayesian kernel machine regression (BKMR) and weighted quantile sum regression (WQS) assessed the relationship between metal mixtures and dyslipidemia.

**Results:**

After adjusting for confounders, lithium (Li) and tin (Sn) were negatively associated with dyslipidemia, with *OR* (*CI*) of 0.654 (0.436, 0.981) and 0.800 (0.649, 0.985), respectively. In BKMR, Li exhibited a negative correlation with dyslipidemia across the whole exposure range. The interactions between Li and Nickel (Ni), Sn and manganese (Mn), and Ni and Mn were found. In WQS, the overall effect of the metal mixtures was not statistically significant, with Li and Sn accounting for 7.6% and 16.8% of the weights in negative direction.

**Conclusions:**

Serum Li and Sn were associated with lower dyslipidemia risk in an observational study of this rural Chinese cohort. Li had an overall negative association with dyslipidemia in the metal mixtures. The potential interactions between metals warrant further investigation.

## Graphical Abstract

**Figure:**
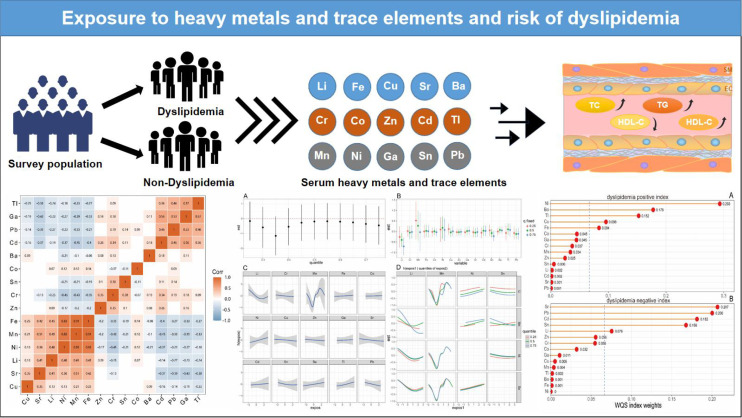

## 1 Introduction

Exposure to metal mixtures is common and can disrupt lipid metabolism and harm health. Mercury, lead, cadmium and arsenic are toxic substances that accumulate in the lungs, kidneys, liver, endocrine and other tissues and organs (1, 2). Studies have shown exposure to these metals alter lipid profiles, increasing total cholesterol (TC), low density lipoprotein cholesterol (LDL-C) and triglycerides (TG), while lowering high density lipoprotein cholesterol (HDL-C) (3). Such lipid changes may lead to cardiovascular disease, non-alcoholic fatty liver disease and atherosclerosis. However, most studies have focused on single metals (4–7). In reality, people are often exposed to multiple metals from pollution, occupation and diet (8, 9). Metals may interact synergistically and cause complex effects. Therefore, investigating relationships between metal mixtures and dyslipidemia is crucial yet understudied.

Previous studies have demonstrated associations between exposure to metals like lead and cadmium and abnormal lipid metabolism. However, few studies have examined the effects of exposure to multiple metal mixtures on lipid metabolism (10, 11). Moreover, interactions between different metal elements may result in more complex biological consequences (12). There is a significant association between the levels of Cd, Cu, Mn, Mo, Ni, Se and Zn in the blood and TG, TC, LDL and high-density lipoproteins result from a Canadian Health Measures Survey. The combined effect of an increase in the interquartile range of heavy metals is positively correlated with the percentage increase of TC and LDL, which are 8.82% (95% CI: 7.06, 10.57), 7.01% (95% CI: 2.51, 11.51), respectively (13). Similarly, evidence suggests the metal mixture of blood Pb, and the essential metals was positively associated with all of the serum lipid profiles. And, an inverse U-shaped association of Pb with Ln TG and the positive interactive effect between blood Pb and Mg levels on TC and LDL-C (14). Interestingly, differing lipid responses to the same metal mixtures have been reported across populations (15). For instance, one study found an inverse association between metals and dyslipidemia in women (16). Although inconsistent, current evidence indicates links between multi-metal exposures and abnormal blood lipids.

Dyslipidemia is a known risk factor for chronic diseases in rural populations where metal mixture exposure is common (17–19). To examine this relationship in rural China, we conducted a nested case-control study in Ningxia Province. We employed a range of statistical analysis methods aimed at exploring the association between exposure to multiple metals and the associations of dyslipidemia. Findings will facilitate interventions for disease management and prevention.

## 2 Materials and methods

### 2.1 Study design and population

This study employed a nested case-control approach. A prospective cohort of individuals aged 24 to 75 years was established between 2008 and 2012 in Qingtongxia City of Wuzhong and Pingluo County of Shizuishan in Ningxia. The inclusion criteria (1), long-term residents (more than 6 months) (2); willingness to provide blood samples and undergo examination (3); signed informed consent (4); no language barriers. The baseline inclusion totaled 2209 participants, from whom questionnaire data and blood samples were collected, and serum elements levels were measured (20). Exposure (serum metal levels) was measured at baseline when all participants were confirmed to be free of dyslipidemia through standardized blood lipid testing.

During a follow-up period in 2019-2020, with a median follow-up duration of 10.5 years, 1655 individuals were successfully followed up, response rate of 74.92%, with questionnaire data and biological samples collected during this period. Among these, 391 cases that developed dyslipidemia during the follow-up were included in the case group. For each incident dyslipidemia case during follow -up, we randomly selected 1 control from cohort members who (1): were alive and dyslipidemia-free at the case’s diagnosis time (index date) (2); were sex -and age - matched (within 3 years) to the case; and (3) had complete serum metal measurements. This approach ensures controls represent the population of cases and preserves the exposure - outcome temporal sequence. All 782 participants in the analytic sample were confirmed dyslipidemia-free at baseline, and those with baseline dyslipidemia were excluded from the original cohort. The detailed recruitment flow chart is presented in **Supplementary Figure 1**. This study was approved by the Ethics Review Board of Ningxia Medical University (Ethics ID 2018-012). All participants provided written informed consent. All investigations were performed in accordance with the tenets of the Declaration of Helsinki.

### 2.2 Demographic and physical information

Demographic information, socioeconomic status, and lifestyle factors, including gender, age, smoking status, alcohol intake, and histories of chronic diseases, were conducted by well-trained investigators face-to-face. Body measurements, including height, weight, and blood pressure. The definition of variables and the method of physical examination are shown in the supplementary materials (**Supplementary Methods**).

### 2.3 Ascertainment of incident dyslipidemia

Venous blood samples were drawn from subjects who had been fasting for at least 8 hours. After collecting, the blood was centrifuged and prepared for examination. Blood lipid profiles, including TC, TG, HDL-C, and LDL-C, were measured using enzymatic assays (CHOD-PAP, Roche diagnosis) and an automatic biochemical analyzer (AU400, Olympus, Japan) (21, 22). Abnormal lipid metabolism was diagnosed according to the Guidelines for Prevention and Treatment of Dyslipidemia in Adults in China (2016 revised edition) (23), based on one or more abnormal lipid indexes (1): High Total Cholesterol group, defined as TC ≥ 6.22 mmol/L (2); high Triglycerides group, defined as TG ≥ 2.26 mmol/L (3); Low high-density lipoprotein cholesterol group, defined as HDL-C < 1.04 mmol/L; and (4) High low-density lipoprotein cholesterol group, defined as LDL-C ≥ 4.14 mmol/L.

### 2.4 Metal elements measurement

We have previously detected 23 elements in serum at baseline and investigated their associations with metabolic syndrome (24). Serum samples were extracted from -80 °C storage and dissolved at 4 °C. For pretreatment, quantified samples were acidified with nitric acid in a microwave digestion tank. Measurements were performed using inductively coupled plasma optical emission spectrometry (ICP-OES) (Varian710ES, America), a well-established technique for multi-elemental analysis, after acid removal, dilution and volume adjustment. Detailed operating procedures and accuracy check are provided in **Supplementary Materials**.

This study selected 15 metal trace elements with sample detection rates above 80% to ensure accurate data for further investigation. The chosen elements are Lithium (Li), Chromium (Cr), Manganese (Mn), Iron (Fe), Cobalt (Co), Nickel (Ni), Copper (Cu), Zinc (Zn), Gallium (Ga), Strontium (Sr), Palladium (Pd), Cadmium (Cd), Tin (Sn), Barium (Ba), and Thallium (Tl). For metal elements with measurements below the limit of detection (LOD), we performed multiple random imputation. Specifically, we generated 5 independent imputed datasets, where each below-LOD value was replaced with a random number drawn from a uniform distribution between LOD/2 and LOD/√2. All analyzes were conducted across the 5 imputed datasets, and results were pooled using Rubin’s rules (11, 25).

### 2.5 Statistical analysis

Demographic and physical characteristics of the dyslipidemia and control groups were summarized using descriptive analyzes. Continuous variables including age, BMI, waist and hip circumferences, and blood lipid indexes were expressed as mean ± standard deviation (SD). Categorical variables were presented as numbers (percentage). Based on distribution types, continuous and categorical variables were compared between groups using t-tests and Chi-squared tests, respectively, to ensure statistical reliability.

Serum trace element concentrations were described using mean ± SD and median (25th, 75th percentiles) and compared between cases and controls using Mann-Whitney U tests. Prior to analysis, serum levels were naturally log-transformed to account for skewed distributions. Pearson’s correlation analysis examined correlations between all 15 trace elements, which were visually represented via a correlation matrix heatmap.

Initially, we employed single-exposure models to assess potential associations between exposure to individual metal elements and dyslipidemia. Using conditional logistic regression with natural log-transformed elements as continuous variables, we calculated crude and adjusted odds ratios (*OR*) and 95% confidence intervals (*CI*). Single-exposure models were rigorously adjusted for potential confounders including body mass index (BMI), smoking status (current/former/never), alcohol consumption (current/former/never) and physical activity (≥3 sessions/week as “yes”, <3 as “no”). These adjustments allowed more accurate evaluation of relationships between metal exposures and dyslipidemia, minimizing influence from other factors. In single-metal analysis, the significance threshold is adjusted through false discovery rate (FDR) correction to q<0.05. The multi-element model analyzed fifteen serum metals, adjusted for BMI, smoking, alcohol consumption, physical activity, drinking tea (yes or no) and education levels (no formal education, primary school, middle school or beyond). Confounders were selected based on three criteria (1): established biological plausibility linking the factor to both metal exposure and dyslipidemia (2); consistent evidence from previous epidemiological studies; and (3) availability of complete data in our study population.

To analyze relationships between multiple metal exposures and dyslipidemia, we utilized Weighted Quantile Sum Regression (WQS) and Bayesian Kernel Machine Regression (BKMR) models. The WQS model calculated the overall effect of multiple metals on dyslipidemia using weighted quantile sum regression, accounting for potential correlations and collective effects between exposures (26). The BKMR model detected nonlinear and interactive effects between metal exposures and outcomes using Bayesian kernel machine regression, identifying potential thresholds and interactions missed by traditional linear models (27). In WQS regression, each metal is assigned a weight representing its relative contribution to the overall mixture effect. Weights range from 0 to 1, and the sum of all weights in each direction (positive or negative) equals 1. A higher weight indicates a stronger association between that metal and the outcome. See **Supplementary Methods** for detailed analytical procedures. Both models were adjusted for covariates including BMI, smoking, alcohol consumption, and physical activity to eliminate confounding and obtain more precise exposure-outcome association estimates. Statistical significance was defined as *P* < 0.05. WQS and BKMR were implemented using the R packages “gWQS” and “bkmr” (version 4.2.3), respectively.

## 3 Results

### 3.1 Characteristics of the study population

The study included 391 participants in the case group and 391 participants in the control group, with an equal distribution of males and females. The mean age of participants was similar in both groups. Compared to controls, cases had a lower percentage of participants who drank tea (45.8% vs. 53.2%) and a higher percentage of illiterate participants (37.3% vs. 42.5%). However, these differences were only statistically significant for drinking tea (*p* = 0.038). There were no significant differences in physical activity levels or BMI categories between cases and controls. Blood lipid indexes showed that cases had higher levels of TC, LDL-C, and lower levels of HDL-C compared to controls, all statistically significant (*p* < 0.001). Current smoking was slightly more prevalent among cases than controls (18.4% vs. 12.8%), but this difference was not statistically significant (*p* = 0.095). There were no significant differences in alcohol consumption, hypertension, or diabetes prevalence between cases and controls. Shown in **Table 1**.

**Table 1:** Basic characteristics of study participants at baseline.

Characteristics	Cases	Controls	*P*-value^a^
	N = 391	N = 391	
Gender, n(%)			
Male	151 (38.6)	151 (38.6)	
Female	240 (61.4)	240 (61.4)	
Age (years)	47.55 ± 9.67	47.79 ± 9.71	
Drinking tea (yes), n(%)	179 (45.8)	208 (53.2)	0.038*
Physical activity (yes), n(%)	16 (4.1)	22 (5.6)	0.318
Education levels			
No formal education	146 (37.3)	166 (42.5)	0.090
Primary school	123 (31.5)	122 (31.2)	
Middle school or beyond	122 (31.2)	103 (26.4)	
BMI (kg/m2)			
Underweight (<18.5)	13 (3.3)	18 (4.6)	0.779
Normal weight (18.5-23.9)	239 (61.1)	232 (59.3)	
Overweight (24.0-27.9)	104 (26.6)	113 (28.9)	
Obesity (≥28.0)	35 (9.0)	28 (7.2)	
Mean ± SD	23.34 ± 3.18	23.03 ± 3.13	0.167
Waist circumferences (cm)	79.52 ± 9.04	79.05 ± 9.55	0.478
Hip circumferences (cm)	91.69 ± 6.29	91.55 ± 5.71	0.743
Blood lipid index (mmol/L)			
TC	3.99 ± 0.67	3.82 ± 0.68	<0.001*
TG	1.09 ± 0.43	1.08 ± 0.44	0.886
HDL-C	1.32 ± 0.23	1.41 ± 0.27	<0.001*
LDL-C	2.19 ± 0.56	1.93 ± 0.55	<0.001*
Smoking, n(%)			
Current smoker	72 (18.4)	50 (12.8)	0.095
Never smoker	312 (79.8)	334 (85.4)	
Former smoker	7 (1.8)	7 (1.8)	
Drinking, n(%)			
Current drinker	32 (8.2)	30 (7.7)	0.500
Never drinker	354 (90.5)	359 (91.8)	
Former drinker	5 (1.3)	2 (0.5)	
Hypertension (yes), n(%)	36 (9.2)	40 (10.2)	0.629
Diabetes (yes), n(%)	4 (1.0)	5 (1.3)	0.737

Continuous variables were presented as mean ± SD. Categorical variables were presented as numbers (percentage). BMI, body mass index; TC, Total Cholesterol; TG, Triglycerides; HDL-C, High-density lipoprotein cholesterol; LDL-C, Low-density lipoprotein cholesterol; ^a^ p-Values were derived from Student’s *t*-tests for continuous variables according to the data distribution, and the chi-square test for the categorical variables; with *, *p*-value < 0.05.

### 3.2 Serum metal elements concentrations and correlation

**Table 2** presents the concentrations of 15 metal elements in serum for cases (n = 391) and controls (n = 391). The results indicate that the levels of Mn, Fe, and Ni were significantly higher in cases compared to controls, while Cr, Zn and Sn were significantly lower in cases compared to controls. However, there were no significant differences between cases and controls in the levels of Li, Co, Cu, Ga, Sr, Cd, Ba, Tl, and Pb. Accuracy check for serum element measurements is shown in **Supplementary Material** **Supplementary Table 1**.

**Table 2:** Distributions of serum metals among the study population at baseline.

Elements	Cases (N = 391)		Controls (N = 391)		*P-*valuea
	Median (25th, 75th)	Mean ± SD	Median (25th, 75th)	Mean ± SD	
7Li (μg/L)	0.73 (0.45, 0.96)	0.77 ± 0.32	0.76 (0.51, 0.97)	0.79 ± 0.36	0.170
52Cr (ng/L)	304.66 (279.60, 502.07)	415.16 ± 170.08	323.62 (281.77, 549.55)	384.86 ± 168.74	0.006*
55Mn (ng/L)	17.01 (5.24, 19.49)	12.91 ± 6.97	15.81 (4.46, 18.50)	14.66 ± 7.27	<0.001**
56Fe (ng/L)	1079.60 (594.89, 1399.00)	891.72 ± 440.61	966.51 (498.33, 1212.90)	1002.77 ± 464.13	<0.001**
59Co (ng/L)	0.57 (0.26, 0.89)	0.58 ± 0.42	0.50 (0.23, 0.87)	0.61 ± 0.43	0.350
60Ni (ng/L)	230.78 (64.31, 270.36)	168.50 ± 111.40	195.88 (40.67, 261.62)	194.08 ± 22.64	0.001*
64Cu (ng/L)	61.63 (54.68, 69.27)	61.12 ± 10.56	60.13 (54.45, 67.61)	62.31 ± 10.59	0.140
65Zn (ng/L)	110.08 (94.91, 122.75)	115.80 ± 23.09	113.75 (100.42, 125.71)	110.83 ± 22.64	0.004*
69Ga (ng/L)	5.10 (2.51, 9.21)	6.28 ± 4.36	5.69 (2.53, 9.35)	6.23 ± 4.64	0.630
87Sr (μg/L)	6.55 (5.67, 7.72)	6.78 ± 1.42	6.64 (5.78, 7.62)	6.75 ± 1.43	0.620
112Cd (μg/L)	0.70 (0.32, 1.25)	0.95 ± 0.69	0.85 (0.38, 1.37)	0.84 ± 0.61	0.079
118Sn (ng/L)	8.30 (4.94, 10.95)	9.16 ± 4.96	8.63 (5.56, 13.04)	8.44 ± 4.96	0.048*
137Ba (ng/L)	9.32 (7.41, 11.64)	9.32± 3.28	9.25 (7.15, 11.37)	9.53 ± 3.22	0.270
204Tl (ng/L)	54.08 (28.68, 96.06)	62.16 ± 41.40	56.81 (26.44, 94.79)	65.10 ± 44.82	0.560
207Pb (ng/L)	7.18 (3.65, 10.65)	8.04 ± 4.88	7.49 (4.32, 11.41)	7.49 ± 4.86	0.099

OD, limit of detection; 7Li, Lithium-7; 52Cr, Chromium-52; 55Mn, Manganese-55; 56Fe, Iron-56; 59Co, Cobalt-59; 60Ni, Nickel-60; 64Cu, Copper-64; 65Zn, Zinc-65; 69Ga, Gallium-69; 87Sr, Strontium-87; 112Cd, Cadmium-112; 118Sn, Tin-118; 137Ba, Barium-137; 204Tl, Thallium-204; 207Pb, Lead-207; a *P*-value were derived from Mann-Whitney U tests; with *, p-value < 0.05; with **, p-value < 0.05; μg/L for Li, Sr, and Cd, and ng/L for all other metals.

**Figure 1:**
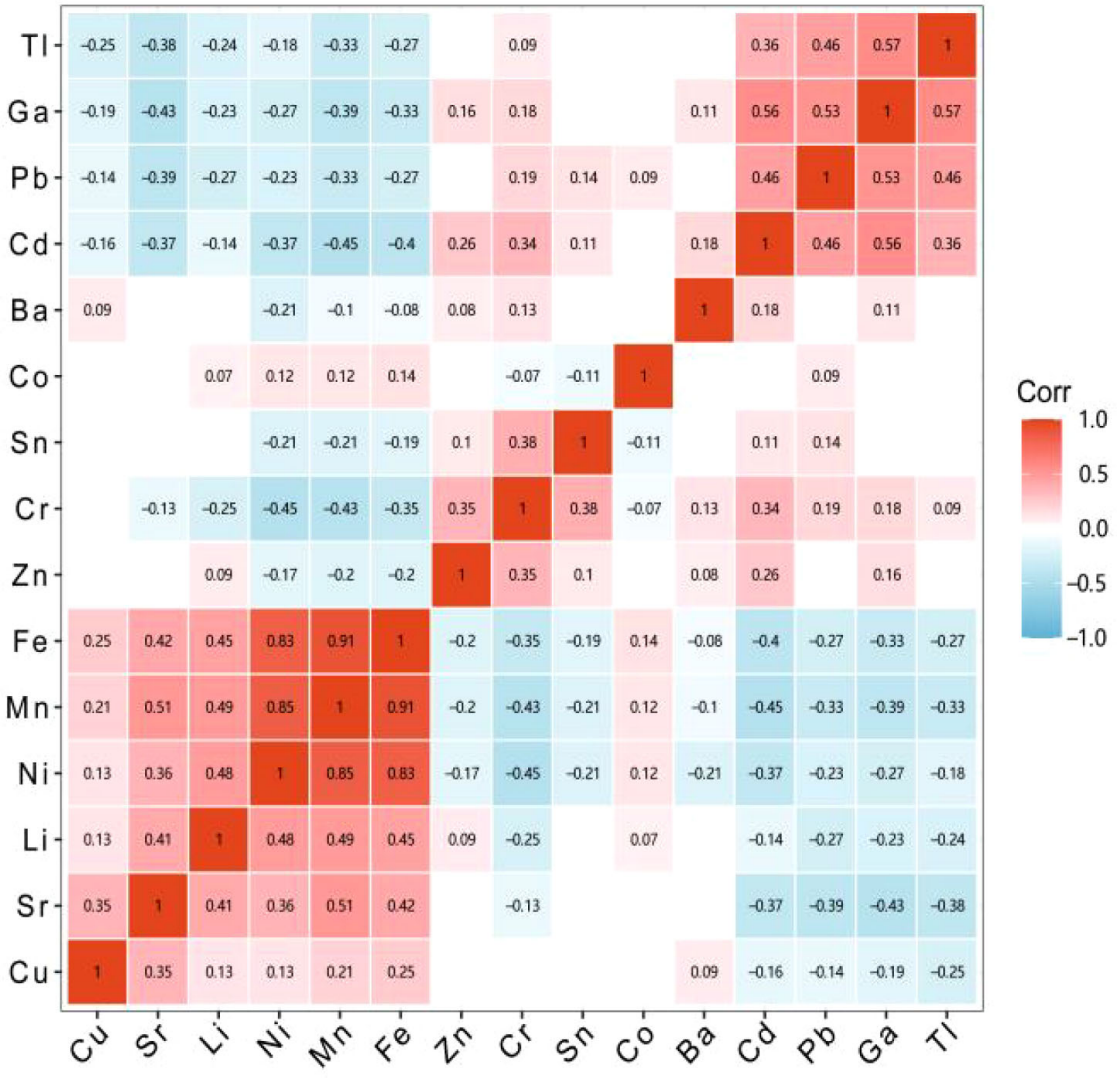
Pearson’s correlation matrix of 15 trace elements among study populations at baseline. Red represents positive correlation, blue represents negative correlation, and grid blank indicates no statistical significance.

The heatmap of correlations illustrated the presence of both positive and negative correlations among fifteen metal elements. Among the positively correlated elements, Mn and Fe exhibited the strongest correlation with a coefficient of 0.91; followed by Mn and Ni, which had a correlation coefficient of 0.85. Regarding negative correlations, both Ni and Cr, as well as Mn and Cd, shared a correlation coefficient of -0.45, as shown in **Figure 1**. In addition, the heatmap suggested that collinearity might exist between Fe, Mn, and Ni.

### 3.3 Association between element and dyslipidemia

The unadjusted results of the conditional logistic regression analysis suggest that there is a correlation between the following elements: Cr, Mn, Ni, Zn, and Sn, and the occurrence of dyslipidemia. The Odds Ratio (*OR*) and its 95% confidence intervals (*CI*) for the elements respectively are as follows: Cr 0.610 (0.430, 0.868), Mn 1.256 (1.043, 1.514), Ni 1.171 (1.030, 1.331), Zn 0.381 (0.155, 0.655), and Sn 0.763 (0.636, 0.915). After adjusting for factors such as BMI, smoking status, alcohol consumption, and physical exercise, only the elements Cr, Zn and Sn show statistical significance. The corresponding *OR* and 95% *CI* are: Cr 0.610 (0.425, 0.877), Zn 0.305 (0.145, 0.638), and Sn 0.742 (0.616, 0.894), as shown in **Table 3**.

**Table 3:** The conditional logistic regression model for the association between metal element and abnormal lipid metabolism.

Elements concentration	*OR* (95%*CI*)		
	Crude model^a^	Adjusted model^b^	Multi-element model^c^
7Li	0.799 (0.597, 1.069)	0.771(0.572, 1.040)	**0.654 (0.436, 0.981)**
52Cr	0.610 (0.430, 0.868)*	0.610 (0.425, 0.877)*	0.700 (0.403, 1.216)
55Mn	1.256 (1.043, 1.514)*	1.254 (1.036, 1.519)	1.429 (0.576, 3.545)
56Fe	1.244 (1.000, 1.498)	1.204 (0.983, 1.475)	1.019 (0.498, 2.081)
59Co	1.024 (0.899, 1.166)	1.016 (0.890, 1.160)	1.016 (0.880, 1.172)
60Ni	1.171 (1.030, 1.331)*	1.172 (1.028, 1.337)	0.903 (0.535, 1.527)
64Cu	1.994 (0.871, 4.565)	1.820 (0.773, 4.289)	2.570 (0.936, 7.059)
65Zn	0.318, (0.155, 0.655)*	0.305 (0.145, 0.638)*	0.480 (0.192, 1.218)
69Ga	1.031 (0.910, 1.168)	1.050 (0.923, 1.193)	1.143 (0.956, 1.193)
87Sr	0.920 (0.478, 1.772)	0.869 (0.446, 1.693)	0.607 (0.244, 1.511)
112Cd	0.885 (0.769, 1.018)	0.887 (0.768, 1.025)	0.990 (0.816, 1.199)
118Sn	0.763 (0.636, 0.915)*	0.742 (0.616, 0.894)*	**0.800 (0.649, 0.985)**
137Ba	1.099 (0.814, 1.485)	1.125 (0.825, 1.534)	1.274 (0.901, 1.802)
204Tl	1.095 (0.947, 1.265)	1.110 (0.950, 1.275)	1.114 (0.927, 1.339)
207Pb	0.918 (0.805, 1.046)	0.905 (0.792, 1.035)	0.855 (0.720, 1.014)

Li, Lithium-7; 52Cr, Chromium-52; 55Mn, Manganese-55; 56Fe, Iron-56; 59Co, Cobalt-59; 60Ni, Nickel-60; 64Cu, Copper-64; 65Zn, Zinc-65; 69Ga, Gallium-69; 87Sr, Strontium-87; 112Cd, Cadmium-112; 118Sn, Tin-118; 137Ba, Barium-137; 204Tl, Thallium-204; 207Pb, Lead-207; ^a^ Crude model was single element expressed by crude odds ratio (95% confidence interval); ^b^ Adjusted model was single element adjusted for body mass index (BMI), smoking status (current, former, or never smoker), alcohol consumption (current, former, or never drinker) and physical exercise; ^c^ Multi-element model included fifteen serum metal elements into analysis and was adjusted for body mass index (BMI), smoking status (current, former, or never smoker), alcohol consumption (current, former, or never drinker), physical exercise (yes or no), drinking tea (yes or no) and education levels (no formal education, primary school, middle school or beyond); *, After false discovery rate (FDR) correction (q < 0.05). Bold indicates statistical significance.

After adjusting for the relevant factors, the multi-element model revealed a significant correlation between only the elements Li and Sn and the occurrence of dyslipidemia, with *OR* and 95% *CI* of 0.654 (0.436, 0.981) and 0.800 (0.649, 0.985), respectively. We used variance inflation factor (VIF) to measure the degree of collinearity of metal mixtures (**Supplementary Table 7**). In the multi-element conditional logistic regression model, we observed severe collinearity between Mn, Ni, and Fe (VIF>10 for Mn and Ni,VIF=9.182 for Fe). Collinearity in traditional regression models leads to unstable coefficient estimates, inflated standard errors, and difficulty in disentangling the independent effects of individual metals. This limitation motivated our use of BKMR and weighted WQS regression, which are specifically designed to handle correlated exposures in mixture analysis.

### 3.4 Multi-element exposures and dyslipidemia

**Figure 2:**
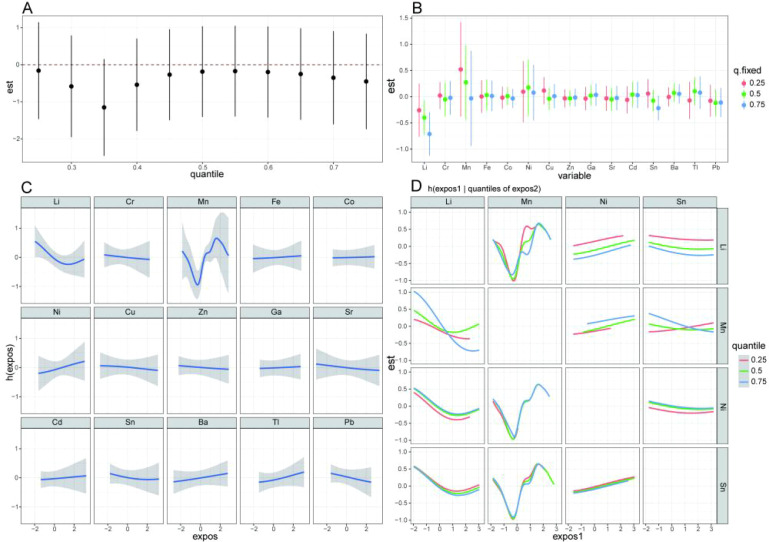
BMKR regression analysis of the effect of metal trace elements mixed exposure on dyslipidemia. BKMR, Bayesian kernel machine regression; model was adjusted for body mass index (BMI), smoking status (current, former, or never smoker), alcohol consumption (current, former, or never drinker) and physical exercise. **(A)** Overall association of the metal mixture with dyslipidemia incidence (difference in the probit of incident dyslipidemia hazard and 95% credibility intervals) when all predictors are at a particular percentile compared with the value when all of them are at their 50th percentile. **(B)** Effects of single trace element (estimates and 95% CI, estimated zero means null), the change in a latent continuous outcome of dyslipidemia when a single trace element is at the 75th vs. 25th percentile, and while all other elements are at their 25th, 50th, or 75th percentiles. “est” is defined as an association between a single trace element and a latent continuous outcome. **(C)** Univariate exposure-response functions (estimates and 95% CI) between exposure to single elements and dyslipidemia when other trace elements are fixed at the 50th percentile. **(D)** Bivariate parallel exposure-metal exposure reaction relationship (difference in the probit of incident dyslipidemia hazard) for a given metal, when a second metal is fixed to the 25th, 50th and 75th percentiles.

We fitted a BKMR model incorporating 15 variable elements to identify important components within the metal mixture and to assess their combined and independent effects on dyslipidemia. The model underwent 10,000 iterations to ensure robust calculation, and its convergence was verified. The results demonstrate that the BKMR model exhibits good convergence performance, as shown in **Supplementary Figure 5**. **Supplementary Figure 2** displays the posterior inclusion probability (PIP) for each metal. Since the PIP for Li, Mn, Ni, and Sn were all greater than 0.6, these variables were of high relative importance in influencing the outcome. **Figure 2A** depicts the collective impact of a 15-metal mixture on the potential binary outcome of dyslipidemia. The findings indicate that as the cumulative level of all metals increases, the joint effect fails to confer statistically significant impact on the occurrence of dyslipidemia. The independent effects of metals reflect the relationship between each metal and dyslipidemia when one metal was present in the 75th percentile and the concentrations of the remaining metals were fixed at the 25th, 50th, or 75th percentiles (95% *CI*). Based on visual inspection, when the other metal concentrations were held at the 50th and 75th percentiles, Li was significantly related to a lower association of dyslipidemia. Please refer to **Figure 2B** for detailed results. **Figure 2C** illustrates the dose-response relationship of individual trace elements, indicating that Li exhibits a ‘ U ‘ shape, Mn displays a wave-like pattern, Cr, Cu and Sr are negatively correlated whereas Ni, Cd and Ba display a positive association.

To reduce the number of interaction tests and minimize multiple comparison issues, we restricted interaction analyzes to metals with PIP > 0.6 in the BKMR model. This threshold was chosen *a priori* to identify metals with the strongest relative importance in the mixture. The interaction curves indicate that there may be interactions among Li, Mn, Sn, and Ni, as shown in **Figure 2D**. To verify the direction and magnitude of interactions, we assessed them and provided statistically significant measures in conditional logistic regression. **Supplementary Table 8** shows that there was an antagonistic effect between Li and Ni (*β* = -1.004, *p* = 0.031), an antagonistic effect between Sn and Mn (*β* = -0.475, *p* = 0.016), and a synergistic effect between Ni and Mn (*β* = 0.091, *p* < 0.001).

To verify the robustness of the BKMR model, we performed a sensitivity analysis. No processing was performed for those samples of metal elements whose measurements were below LOD. The BKMR model was again applied for analysis. The results of sensitivity analyzes were consistent with the main results, confirming the robustness of the results. **Supplementary Figure 3** for detailed results.

**Figure 3:**
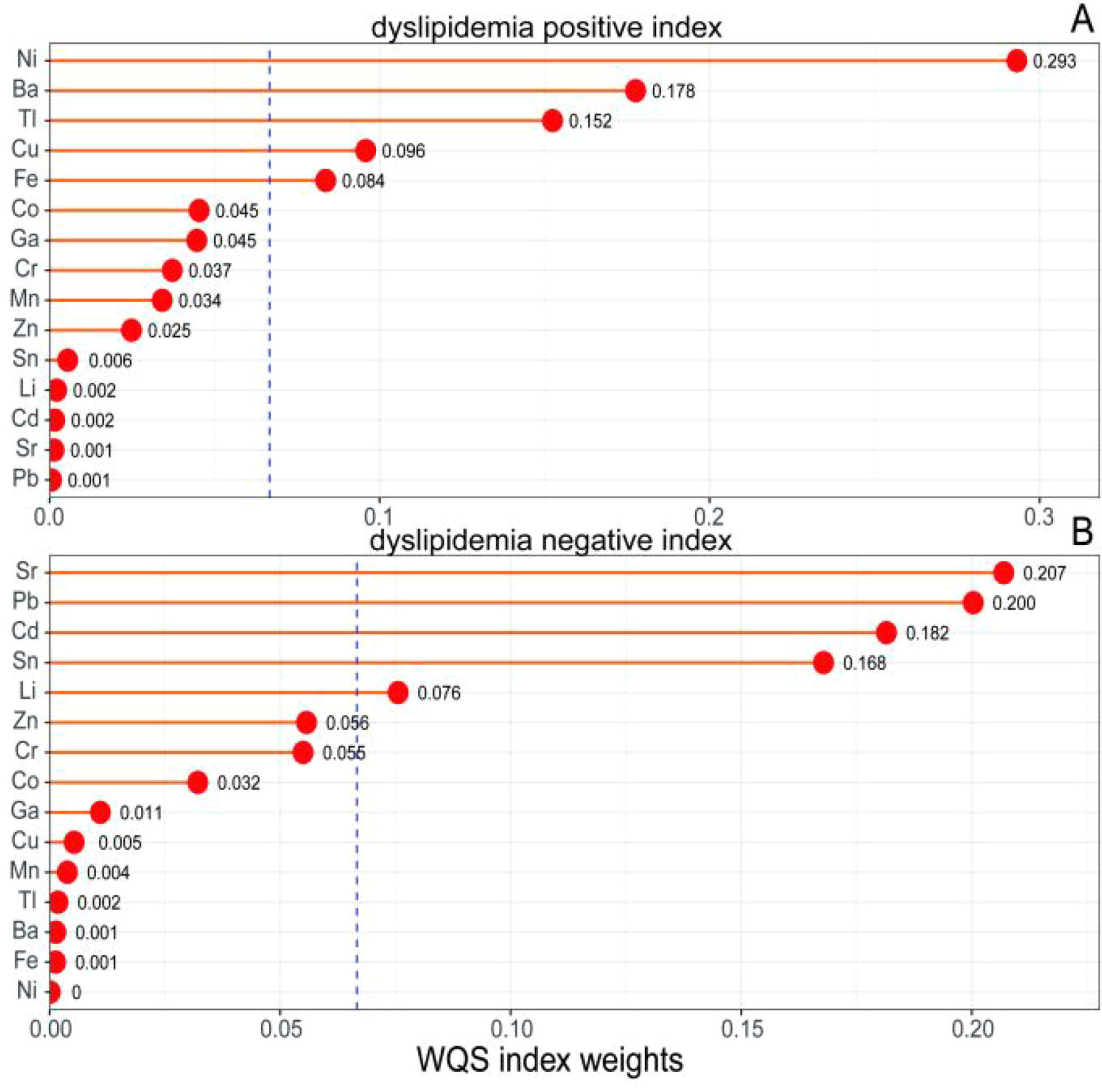
WQS model regression index weights for dyslipidemia. **(A)** WQS index weights of the positive effect of elements on dyslipidemia. **(B)** WQS index weights of the negative effect of elements on dyslipidemia. The blue dotted line represents a weight that exceeds the reference standard (where the weight is more than 1/15 of the reference standard). Model was adjusted for body mass index (BMI), smoking status (current, former, or never smoker), alcohol consumption (current, former, or never drinker) and physical exercise. The blue dashed line represents the average weight (1/15 ≈ 0.067), and metals with weights exceeding this threshold are considered the primary contributors to the mixture effect in that direction.

In addition, we fitted the WQS regression model to further explain the stable prediction of the effect of co-exposure of metal trace elements on dyslipidemia. **Supplementary Table 2** shows the results of WQS regression analysis of the relationship between metal mixtures and dyslipidemia. It shows that WQS is not statistically significant in both positive and negative directions. The model was adjusted according to BMI, smoking status, drinking, physical exercise, and history of coronary heart disease. The weight values of the elements are shown in **Figure 3**. In the positive direction, the weight value of Ni is the largest (0.293), followed by Ba (0.178), Tl, Cu, and Fe are more than 1/15. In the negative direction, the weight of Sr is the largest (0.207), followed by Pb (0.200), Cd, Sn, and Li are more than 1/15.

We performed further WQS regression on TC, TG, HDL-C, and LDL-C, and their results are presented in **Supplementary Table 3**-**S6**. Among all WQS models, only the relationship between metal elements and HDL-C was statistically significant. The estimated wqs index value was 0.101 (*p* = 0.005) in the positive direction. Conversely, the estimated wqs index value was -0.155 (*p* < 0.001) in the negative direction, as listed in **Supplementary Table 5**. The weight values of single element across different blood lipid parameters, as shown in **Supplementary Figure 4**.

## 4 Discussion

In this study, we utilized three analytical methods, multivariate conditional Logistic regression, BKMR model, and WQS regression, to investigate the association between dyslipidemia and metal mixture exposure. Multivariate conditional Logistic regression helped us to quantitatively assess the independent association of each metal element with health risk. The BKMR model allows us to simultaneously account for potential interactions and nonlinear effects between all metal elements. The WQS regression provides us with a quantitative means to identify the elements of the mixture that have the most significant effect on health risk. The combination of these methods enables a more comprehensive understanding and evaluation of the health effects of metal mixture exposure.

The inconsistencies across models reflect different statistical objectives and strengths, and their results are complementary. Conditional logistic regression evaluates each metal’s independent effect while adjusting for others, is sensitive to collinearity, and identified Li and Sn as the only metals with significant inverse associations with dyslipidemia. BKMR assesses the overall mixture effect, nonlinear relationships, and interactions. The non-significant overall effect may be due to opposing effects of different metals. WQS decomposes the mixture effect and found the overall effect non-significant for the same reason as BKMR. Li (7.6%) and Sn (16.8%) contributed most to the negative effect, while Ni (29.3%) contributed most to the positive effect. Importantly, all three models agreed that Li and Sn are inversely associated with dyslipidemia, and Ni is positively associated with it in this population.

The U-shaped dose-response relationship for Li suggests that its association with dyslipidemia is nonlinear: at low concentrations (within the range observed in our study population, median 0.75μg/L), Li is inversely associated with dyslipidemia, but at higher concentrations, this association may reverse. This is consistent with the hormesis theory, which posits that many substances have beneficial effects at low doses but toxic effects at high doses (28, 29). In addition, our study also identified a potential antagonism between Li and Ni. This suggests that Ni will reduce the protective effect of Li on dyslipidemia through antagonism.

Sn is one of the earliest elements discovered by humans. Although Sn is not an essential trace element for maintaining normal physiological functions, it has a potential impact on human health (30). Our results showed the median serum Sn concentration was relatively low at 8.38 (5.36, 11.42) ng/L in all study objects. Thus, only a protective effect of Sn on dyslipidemia was observed, with an OR of 0.800 (0.649, 0.985). The biological mechanisms underlying the observed inverse associations of Li and Sn with dyslipidemia remain unclear. Limited previous studies have suggested that Li may modulate oxidative stress and inflammatory pathways (29), while Sn may be involved in certain metabolic processes (31). However, these hypotheses require confirmation in mechanistic studies, and the potential harmful effects of high-level Li and Sn exposure must be carefully considered.

Mn is closely related to human physiology and greatly impacts nervous system function (32). Mn deficiency can cause embryonic dysplasia, stunted growth and development, dwarfism, and even deformities (33). Mn also affects hematopoietic and metabolic functions (34). Although our multivariate results did not find an independent effect of Mn on dyslipidemia, single factor analysis suggested Mn increased dyslipidemia by 1.254 (1.036, 1519). The exposure-response relationship exhibited an ‘S’-shaped curve, with decreasing then increasing, as shown in **Figure 2C**. Prior studies have reported Mn can increase dyslipidemia risk, with a 7% increase per 1 ng/dL rise in serum Mn (35). A U-shaped association between urinary Mn and metabolic syndrome was described (36). Mn supplementation reduced cholesterol in calcium-deficient ovariectomized rats (37), consistent with the first downward trend we observed. Mn enters the body primarily through diet and environmental exposure. As our site is near a Mn factory, exposure may be increased.

We chose serum as our exposure biomarker because it is minimally invasive, widely used in epidemiological studies, and allows for simultaneous measurement of multiple metals. For non-volatile, non-lipophilic metals such as Li, Sn, and Mn, serum levels reflect both recent (days to weeks) and medium-term (months) exposure. In populations with relatively stable environmental exposure patterns (such as rural residents with consistent dietary habits, living environments, and occupational exposures), serum metal concentrations show good intra-individual reproducibility over time and can serve as a reasonable proxy for long-term exposure (11, 25, 38). Additionally, previous cohort studies using the same study population have demonstrated that baseline serum metal levels are significantly associated with metabolic syndrome (24), further supporting the validity of our exposure assessment approach. Future studies using multiple biomarkers (e.g., urine, hair, nails) would provide a more comprehensive assessment of metal exposure over different time windows.

Additionally, WQS regression results showed Ni had the largest weight in the positive direction for the metal mixture effect on dyslipidemia, indicating Ni was the potential contributor to dyslipidemia risk. This was consistent with single factor analysis, which gave an OR of 1.172 (1.028, 1.337) for Ni. Complex metal-dyslipidemia interactions exist (39). There was a significant antagonistic effect between Mn and Sn, and there was a synergistic effect between Mn and Ni to increase the association of dyslipidemia, as shown in **Figure 2D** and **Supplementary Table 8**.

This study measured the stable isotopes of 15 metal elements in serum, including Li, Cr, Mn, etc., which are not radioactive, i.e., they do not have half-life. Due to fluctuations in dietary intake, fasting status significantly affects lipophilic metals (38). Consequently, all participants in this study were sampled after at least 8 hours of fasting to minimize variability. The serum levels of Li and Sn observed in this study reflect different exposure pathways of public health relevance. Sources of Li include the leakage of lithium into groundwater from the environment, industrial activities related to lithium-ion batteries, and the use of medications (40). Exposure to Sn originates from the consumption of canned foods, occupational inhalation in the welding and electronics manufacturing industries, and the residues of organotin compounds in consumer products, despite these organotin compounds being banned by regulatory authorities (41).

Although all participants were free of dyslipidemia at baseline according to clinical cutoffs, there was substantial variation in lipid levels within the normal range. Cases had significantly higher baseline TC, LDL-C, and lower HDL-C levels than controls, all within the normal reference range. These differences likely reflect subclinical lipid abnormalities that precede the development of clinical dyslipidemia, consistent with the progressive nature of lipid metabolism disorders.

Our study has several strengths. First, the nested case-control design matched subjects by age and gender, reducing confounding. Second, this design provides stronger evidence for temporal associations compared to cross-sectional studies. Third, utilizing different mixture modeling approaches to analyze metal-dyslipidemia relationships may mitigate collinearity issues and enhance reliability (42). The BKMR method enables quantitative exposure-response assessment and precise prediction of associations between environmental pollutants and dyslipidemia, providing robust supportive evidence. Meanwhile, WQS can directly reflect influences of pollutants at different levels (43, 44). However, there are some limitations in this study. The nested case-control design has weaker causal inference compared to cohort studies (45). The relatively high mean participant age (47.67 years) indicates lower metal exposure levels than younger populations (46). A single baseline measurement cannot capture dynamic changes in metal exposure over the long follow-up period, which may introduce non-differential measurement error and bias effect estimates toward the null. Moreover, serum elements are influenced by metabolites and oxidative stress (47). Despite rigorous adjustment for known confounders, residual confounding remains an important limitation of this observational study. Unmeasured or incompletely measured factors may influence our results, including (1): detailed dietary patterns (e.g., intake of saturated fat, fiber, and dietary supplements containing trace elements) (2); genetic predispositions (e.g., polymorphisms in lipid metabolism genes such as APOE) (3); medication use (e.g., lipid-lowering drugs, antihypertensives, and lithium-containing medications) (4); chronic psychological stress and sleep disorders. These factors may independently affect lipid metabolism and metal exposure levels, and future large-scale studies should collect comprehensive data on these variables to improve confounding control.

Overall, our study provides insights into the potential impacts of multiple trace elements on dyslipidemia and underscores the importance of considering combined exposures when assessing health risks related to trace elements. Future studies require large-scale, high-quality cohort that account for dietary exposures to elucidate the mechanisms of multi-element co-exposures on the occurrence and progression of dyslipidemia.

## 5 Conclusions

Serum Li and Sn levels alone were negatively correlated with dyslipidemia in this rural population in a nested case-control study in Northwest China. Among them, Li showed an overall negative association with dyslipidemia in the multiple heavy metal mixture model analysis. We found an antagonistic effect between Li and Ni, an antagonistic effect between Sn and Mn, and a synergistic effect between Ni and Mn were found. In general, this study provided suggestive evidence of an inverse association between Li and dyslipidemia, but the underlying mechanism of the interaction between metals needs to be further explored in future prospective cohort studies with a large sample size to strengthen the validity of the findings.

## Data Availability

The original contributions presented in the study are included in the article/**Supplementary Material**. Further inquiries can be directed to the corresponding authors.
