# Supplementary material for: Exposure to heavy metals and trace elements and risk of dyslipidemia: a nested case-control analysis in rural adults
Source: Front Endocrinol (Lausanne). 2026 May 21;17:1851531. doi: 10.3389/fendo.2026.1851531 (PMC13233187; doi:10.3389/fendo.2026.1851531)
Supplement: Supplementary file 1 [file DataSheet1.docx]

**SUPPLEMENTARY MATERIAL**

**Supplementary methods**

***Definition of variables***

Smoking was defined as smoking more than 1 cigarette per day or accumulated over a lifetime, alcohol used was defined as having consumed a variety of alcohol in the past year or at least once a week, and physical activity was defined as exercising more than 30 minutes at least three times a week or 150 minutes a week in your spare time. Hypertension was defined as measured values ≥ 140 mmHg for SBP or ≥ 90 mmHg for DBP, self-reported physician diagnosis, or reported use of antihypertensive medication. Diabetes was defined as fasting glucose ≥7.0 mmol/L, self-reported physician diagnosis, or antidiabetic medication use (insulin or oral hypoglycemic agents).

***The method of physical examination***

Blood pressure were taken from the subjects in the morning while they were fasting and wearing light clothing. Height and weight were determined using a weight scale (Omron, China). Body mass index (BMI) was calculated using weight and height to assess obesity. Blood pressure was measured three times in the right arm while participants were resting and seated, using an electronic sphygmomanometer (Omron HEM 7301-IT, China).

***WQS regression model specification***

WQS is a statistical model for conducting multivariate regression analysis on high-dimensional datasets. It is frequently used to explore the relationship between exposure to environmental pollutants and health outcomes. By constructing a weighted exposure index, WQS tests the association between this index and the outcome to estimate the overall effect of multiple exposure variables on the outcome.

The model is as follows:

$$g(\mu) = \beta_{0} + \beta_{1}(\sum_{i=1}^{c} \omega_{i}q_{i}) + z\phi$$

$$WQS = \sum_{i=1}^{c} \omega_{i}q_{i}$$

$g(\mu)$: Link Function of Generalized Linear Models;

$\beta_{0}$: Intercept;

*C*: Number of elements Exposures;

$\omega$: Exposure weights, where the weight w of each element is less than 1, and the sum of the weights w of all elements equals 1;

*q*: The scoring is based on quartiles, with each element concentration falling into four groups: q1, q2, q3, and q4. Points are assigned as follows: 0 for q1, 1 for q2, 2 for q3, and 3 for q4;

$\beta_{1}$: Coefficient of sum of quantiles of elements;

*z*: Covariate matrix;

$\phi$: Covariate coefficient.

The WQS regression model was implemented using the R package "gWQS" (version 3.0.0). We used 1000 bootstrap samples to estimate the weights and their standard errors. A 70/30 training/validation split was used to avoid overfitting, and we performed 10 repeated holdout validations to assess model stability. All metal concentrations were quantiled into quartiles prior to analysis. The model was adjusted for the same set of covariates as the other analyses. Two WQS regressions were performed in our actual data analysis to test the positive and negative associations between the mixture of element exposures and dyslipidemia. The weights were estimated using bootstrap resampling. We fitted a link function based on dichotomous outcomes and empirical estimation of weights, with weights ranging from 0 to 1 and summing to 1 within each group. Ultimately, we evaluated the magnitudes and statistical significances of the correlations among each group, dyslipidemia, and the trace element weights within their respective groups.

***BKMR regression model specification***

The BKMR model has been widely applied in studies on the relationship between environmental mixtures exposure and health. The BKMR model can help us identify the non-linear and interactive effects between environmental exposure and health. It can estimate the impact of multiple environmental exposures simultaneously without the need for multiple comparison correction. In addition, it can take into account potential confounding factors and effect modifiers, and provide information on the size and direction of the effect of each environmental exposure through the posterior distribution[https://doi.org/10.1093/biostatistics/kxu058]. The specific formula is as follows:

$$Y_{i}=h(z_{i},...,z_{im})+x_{i}\beta+\epsilon_{i}$$

*Y*: Individual i dyslipidemia event;

*z*: The MTh element that has been exposed;

*h* (): The unknown exposure response function;

$\beta$: The effect of covariates;

$\epsilon$: Residual.

The BKMR model was implemented using the R package "bkmr" (version 0.2.0). We used a Gaussian kernel with default prior specifications for the variance components (inverse-gamma priors with shape = 0.01 and rate = 0.01). All metal concentrations were standardized to have mean 0 and standard deviation 1 prior to analysis. We ran the model for 50,000 iterations, with the first 40,000 iterations discarded as burn-in, and thinned the remaining 10,000 iterations by 10 to reduce autocorrelation. Convergence was assessed using the Gelman-Rubin R-hat statistic, with all parameters having R-hat < 1.05 indicating good convergence. For the binary dyslipidemia outcome, we used a probit link function. We employed spike-and-slab variable selector to obtain posterior inclusion probabilities (PIP) for each metal. Metals with PIP values greater than 0.6 were considered important. Additionally, when a single metal was at its 25th percentile to 75th percentile, we analyzed the single-exposure effects of that metal on dyslipidemia risk within the mixture, while the other metals were held at their corresponding percentiles.

***Methodological description of the elemental analysis of serum***

1. Instruments and reagents required for measurement

Inductively coupled plasma emission spectrometer (Varian710ES), Milli-Q pure water machine (Millipore Corporation, USA), graphite furnace, MDS-8 microwave digestion instrument (Shanghai Xinyi Microwave Chemical Technology Co., LTD.) multi-element mixed standard solution (Merck, Germany), nitric acid (superior grade pure), perchloric acid (superior grade pure) purchased from Beijing Chemical Plant.

1. Sample collection and processing

The collected serum samples were frozen at -80℃ immediately after isolation to prevent changes in elemental content. Prior to measurements, samples were thawed according to standard procedures and prepared by microwave digestion. The process is as follows: The serum sample to be tested was dissolved at room temperature, mixed by swirl shaking, and 500ul of the serum sample was placed in a 20mL digestion tank of perfluoroalkoxy material (the digestion tank was soaked in 30%-40% nitric acid overnight, and removed and cleaned with deionized water before use). Then 3ml of nitric acid and 7ml of pure water were added to it, and the lid was tightened. After the digestion is completed and the temperature in the furnace drops to 100℃, the digestion tank is taken out and placed in the fattening kitchen. After the temperature of the digestion tank drops to room temperature, loosen the vent valve with a wrenter to let off the air, and transfer the digestion solution to a 10mL plug cuvette with deionized water (repeated three times). Add 80ul of rhodium standard solution at 800, and constant volume to 8ml to be tested. Microwave digestion instrument temperature control digestion program as follows:

Table 1 Microwave digestion instrument temperature control digestion program

| Step | Temperature (℃) | Time (min) | Power (pa) |
| --- | --- | --- | --- |
| 1 | 120 | 5 | 1000 |
| 2 | 150 | 3 | 1000 |
| 3 | 180 | 10 | 1000 |

1. ICP-OES analysis

Preparation of standard series: Take 0.1 ml, 0.2ml, 0.4 ml, 0.8 ml, 1.6 ml of 100ug/ml multi-element mixed standard solution in 100ml volumetric flask, and volume to 100ml with 1% HNO3. The concentrations of the standard series were 0.1ug/ml, 0.2 ug/ml, 0.4ug/ml, 0.8ug/ml and 1.6ug/ml, respectively.

The working conditions of ICP-OES were as follows: the transmission power was 1.00 kw, the plasma flow rate was 15.0 L/min, and the auxiliary flow rate was 1.5(L/min).

4. Accuracy check

Detection limit: The blank solution was used for 10 parallel determinations, and the element concentration corresponding to 3 times the standard deviation was the detection limit.

Precision: The same sample was injected 10 times in a row, and the relative standard deviation (RSD) of each element was calculated.

Recovery rate of standard addition: two samples of the same sample were taken, one of which was added with quantitative standard material of the components to be tested; Two samples were analyzed at the same time according to the same analytical steps. The ratio of the difference between the result of the spiked sample and the theoretical value of the added standard substance was the recovery rate of the sample. The specific results are shown in **Table S1.**

**Reference**

Yu, L., Liu, W., Wang, X., Ye, Z., Tan, Q., Qiu, W., Nie, X., Li, M., Wang, B., Chen, W., 2022. A review of practical statistical methods used in epidemiological studies to estimate the health effects of multi-pollutant mixture. Environ Pollut 306, 119356. https://doi.org/10.1016/j.envpol.2022.119356

**Table of Contents**

**Fig. S1** Flowchart for participant recruitment from the present study.

**Table S1** Accuracy check for serum element measurements.

**Fig. S2.** Posterior Inclusion Probability of the BKMR model.

**Fig. S3** Sensitivity analysis of BMKR regression analysis of the effect of metal trace elements mixed exposure on dyslipidemia.

**Table S2** Results from the WQS regression analysis for examining the link between metal mixtures and dyslipidemia.

**Table S3** Results from the WQS regression analysis for examining the link between metal mixtures and TC.

**Table S4** Results from the WQS regression analysis for examining the link between metal mixtures and TG.

**Table S5** Results from the WQS regression analysis for examining the link between metal mixtures and HDL-C.

**Table S6** Results from the WQS regression analysis for examining the link between metal mixtures and LDL-C.

**Fig. S4** WQS model regression index weights for TC, TG, HDL-C and LDL-C.

**Table S7** Collinearity Statistics.

**Fig. S5** Validation of the convergence of the BKMR model.

**Table S8** Interactions between metal elements in conditional logistics regression.

**
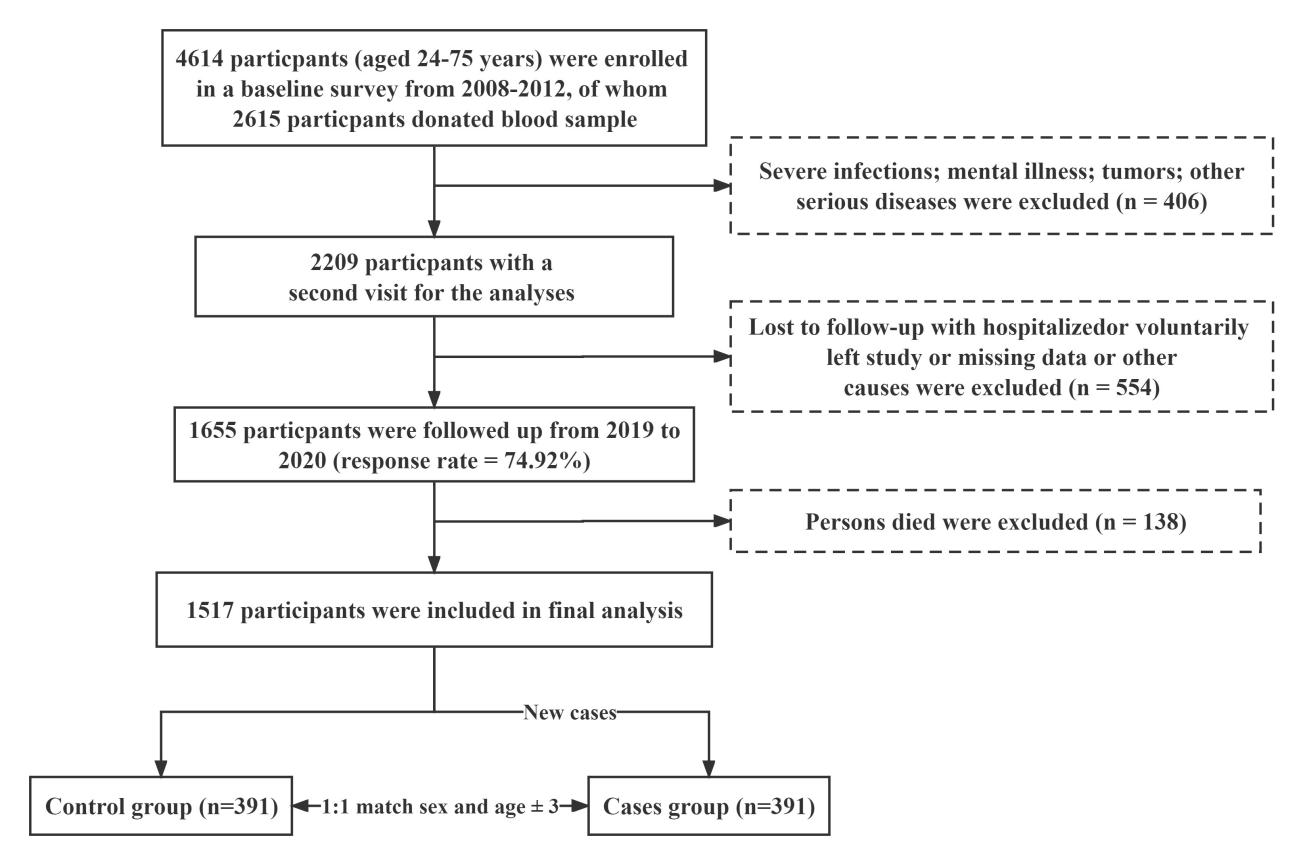
**

**Fig. S1** **Flowchart for participant recruitment from the present study.** Control group, Non-dyslipidemia group; Cases group, dyslipidemia group; Inclusion and Exclusion, diagnosed according to the Guidelines for Prevention and Treatment of Dyslipidemia in Adults in China (2016 revised edition).

**Table S1 Accuracy check for serum element measurements**

| Elements | LOD | < LOD, n (%) | RSD (%) | Adding standard recovery (%) |
| --- | --- | --- | --- | --- |
| 7Li (μg/L) | 0.22 | 18 (2.30) | 2.31 | 92 |
| 52Cr (ng/L) | 24.05 | 0 (0) | 1.22 | 106 |
| 55Mn (ng/L) | 0.32 | 0 (0) | 1.83 | 89 |
| 56Fe (ng/L) | 115.87 | 0 (0) | 1.23 | 94 |
| 59Co (ng/L) | 0.01 | 8 (1.02) | 2.99 | 99 |
| 60Ni (ng/L) | 2.02 | 2 (0.26) | 1.70 | 101 |
| 64Cu (ng/L) | 11.58 | 0 (0) | 1.46 | 104 |
| 65Zn (ng/L) | 35.15 | 0 (0) | 1.74 | 97 |
| 69Ga (ng/L) | 1.03 | 79 (10.10) | 3.05 | 101 |
| 87Sr (μg/L) | 1.02 | 0 (0) | 1.17 | 117 |
| 112Cd (μg/L) | 0.06 | 25 (3.20) | 3.47 | 108 |
| 118Sn (ng/L) | 0.41 | 9 (1.15) | 1.46 | 96 |
| 137Ba (ng/L) | 2.27 | 20 (2.56) | 1.30 | 97 |
| 204Tl (ng/L) | 1.09 | 10 (1.28) | 3.34 | 103 |
| 207Pb (ng/L) | 0.11 | 6 (0.77) | 2.89 | 96 |

Abbreviations: LOD, limit of detection; 7Li, Lithium-7; 52Cr, Chromium-52; 55Mn, Manganese-55; 56Fe, Iron-56; 59Co, Cobalt-59; 60Ni, Nickel-60; 64Cu, Copper-64; 65Zn, Zinc-65; 69Ga, Gallium-69; 87Sr, Strontium-87; 112Cd, Cadmium-112; 118Sn, Tin-118; 137Ba, Barium-137; 204Tl, Thallium-204; 207Pb, Lead-207.

**
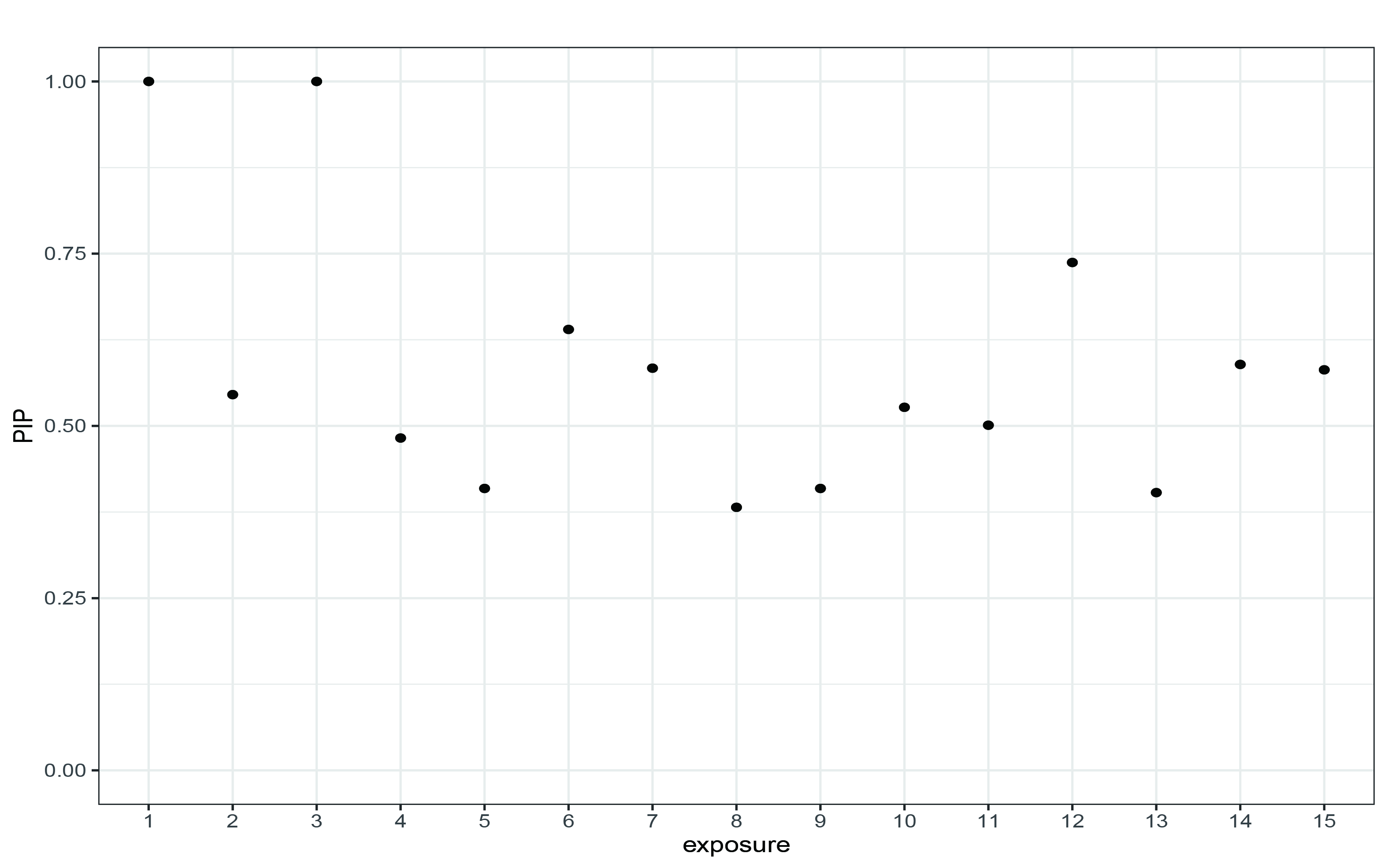
**

**Fig. S2** **Posterior Inclusion Probability of the BKMR model.** PIP, Posterior Inclusion Probability; exposure, 1-15 refers to the fifteen trace elements; 1, Li; 2, Cr; 3, Mn; 4, Fe; 5, Co; 6, Ni; 7, Cu; 8, Zn; 9, Ga; 10, Sr; 11, Cd; 12, Sn; 13, Ba; 14, Tl; 15, Pb. Model was adjusted for body mass index (BMI), smoking status (current, former, or never smoker), alcohol consumption (current, former, or never drinker) and physical exercise.

**
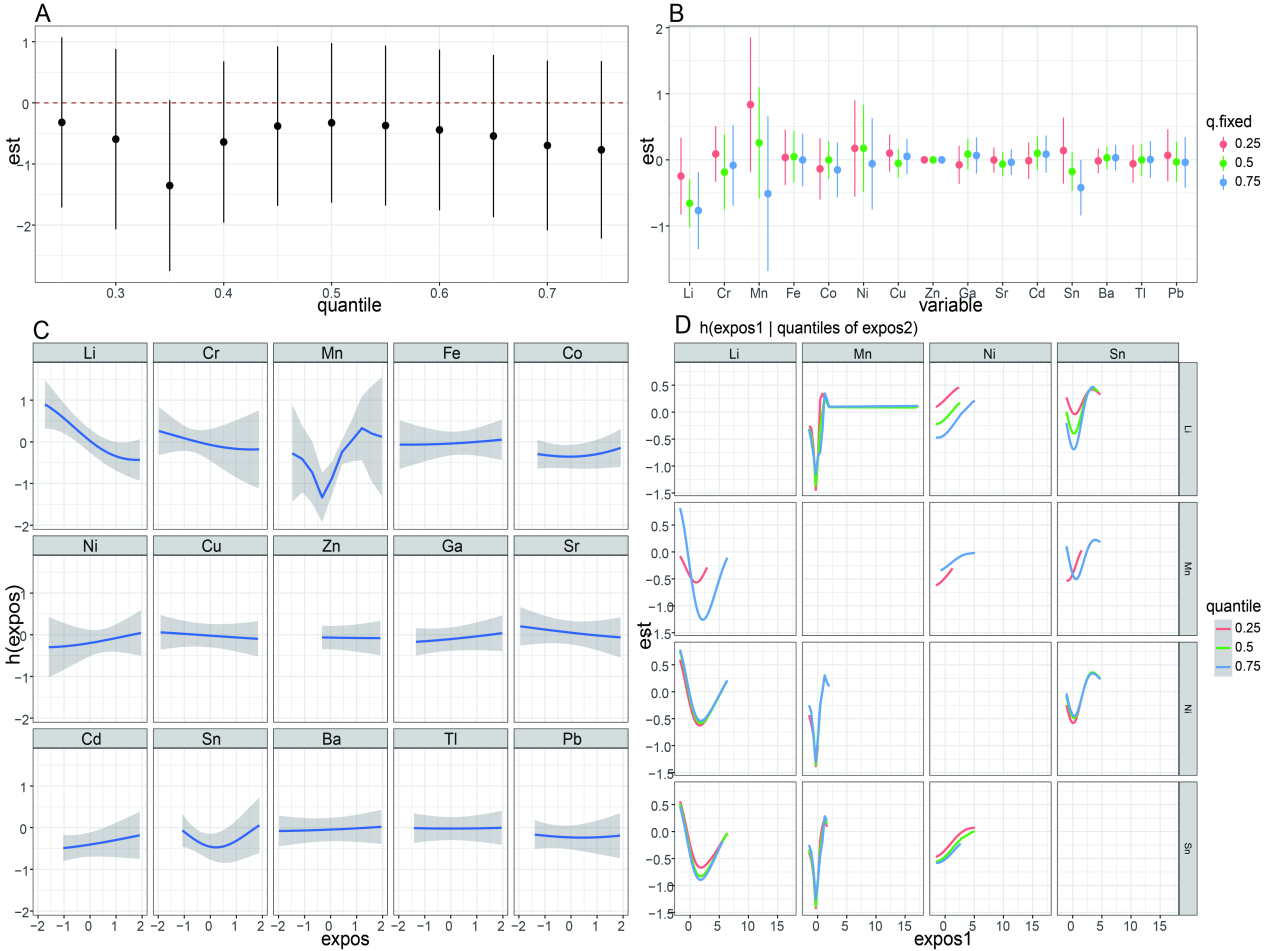
**

**Fig. S3 Sensitivity analysis of BMKR regression analysis of the effect of metal trace elements mixed exposure on dyslipidemia.** BKMR, Bayesian kernel machine regression; Model was adjusted for body mass index (BMI), smoking status (current, former, or never smoker), alcohol consumption (current, former, or never drinker) and physical exercise. **(A)** Overall association of the metal mixture with dyslipidemia incidence (difference in the probit of incident dyslipidemia hazard and 95% credibility intervals) when all predictors are at a particular percentile compared with the value when all of them are at their 50th percentile. **(B)** Effects of single trace element (estimates and 95% CI, estimated zero means null), the change in a latent continuous outcome of dyslipidemia when a single trace element is at the 75th vs. 25th percentile, and while all other elements are at their 25th, 50th, or 75th percentiles. “est” is defined as an association between a single trace element and a latent continuous outcome. **(C)** Univariate exposure-response functions (estimates and 95% CI) between exposure to single elements and dyslipidemia when other trace elements are fixed at the 50th percentile. **(D)** Bivariate parallel exposure-metal exposure reaction relationship (difference in the probit of incident dyslipidemia hazard) for a given metal, when a second metal is fixed to the 25th, 50th and 75th percentiles.

**Table S2 Results from the WQS regression analysis for examining the link between metal mixtures and dyslipidemia**

| Indicator | Positive index | |  | Negative index | |
| --- | --- | --- | --- | --- | --- |
|  | Estimate | *P* |  | Estimate | *P* |
| intercept | -0.703 | 0.566 |  | 0.253 | 0.841 |
| wqs | -0.287 | 0.152 |  | -0.369 | 0.076 |
| Smoking | -0.652 | 0.011 |  | -0.699 | 0.007 |
| Drinking | 0.163 | 0.623 |  | 0.227 | 0.496 |
| Physical activity | 0.335 | 0.446 |  | 0.268 | 0.545 |
| BMI | 0.022 | 0.444 |  | 0.026 | 0.357 |

Abbreviations: Weights of WQS regression models were assigned to each metal element in positive direction and negative direction; Model was adjusted for body mass index (BMI), smoking status (current, former, or never smoker), alcohol consumption (current, former, or never drinker) and physical exercise.

**Table S3 Results from the WQS regression analysis for examining the link between metal mixtures and TC**

| Indicator | Positive index | |  | Negative index | |
| --- | --- | --- | --- | --- | --- |
|  | Estimate | *P* |  | Estimate | *P* |
| intercept | 4.210 | 0.000 |  | 4.040 | 0.000 |
| wqs | -0.118 | 0.190 |  | -0.009 | 0.932 |
| Smoking | 0.226 | 0.048 |  | 0.242 | 0.033 |
| Drinking | 0.023 | 0.878 |  | 0.017 | 0.908 |
| Physical activity | 0.170 | 0.392 |  | 0.162 | 0.418 |
| BMI | 0.003 | 0.827 |  | 0.003 | 0.839 |

Abbreviations: Weights of WQS regression models were assigned to each metal in positive direction and negative direction; TC, Total cholesterol; Model was adjusted for body mass index (BMI), smoking status (current, former, or never smoker), alcohol consumption (current, former, or never drinker) and physical exercise.

**Table S4 Results from the WQS regression analysis for examining the link between metal mixtures and TG**

| Indicator | Positive index | |  | Negative index | |
| --- | --- | --- | --- | --- | --- |
|  | Estimate | *P* |  | Estimate | *P* |
| intercept | 1.190 | 0.051 |  | 1.040 | 0.096 |
| wqs | -0.005 | 0.966 |  | 0.098 | 0.398 |
| Smoking | -0.046 | 0.711 |  | -0.038 | 0.759 |
| Drinking | -0.217 | 0.180 |  | -0.227 | 0.161 |
| Physical activity | 0.099 | 0.647 |  | 0.114 | 0.598 |
| BMI | 0.034 | 0.017 |  | 0.033 | 0.020 |

Abbreviations: Weights of WQS regression models were assigned to each metal in positive direction and negative direction; TG, Triglyceride; Model was adjusted for body mass index (BMI), smoking status (current, former, or never smoker), alcohol consumption (current, former, or never drinker) and physical exercise.

**Table S5 Results from the WQS regression analysis for examining the link between metal mixtures and HDL-C**

| Indicator | Positive index | |  | Negative index | |
| --- | --- | --- | --- | --- | --- |
|  | Estimate | *P* |  | Estimate | *P* |
| intercept | 1.800 | 0.000 |  | 2.160 | 0.000 |
| wqs | **0.101** | **0.005** |  | **-0.155** | **0.000** |
| Smoking | 0.004 | 0.943 |  | -0.009 | 0.872 |
| Drinking | -0.018 | 0.808 |  | 0.002 | 0.981 |
| Physical activity | 0.099 | 0.314 |  | 0.074 | 0.450 |
| BMI | -0.015 | 0.026 |  | -0.012 | 0.054 |

Abbreviations: Weights of WQS regression models were assigned to each metal in positive direction and negative direction; HDL-C, high-density lipoprotein cholesterol; Model was adjusted for body mass index (BMI), smoking status (current, former, or never smoker), alcohol consumption (current, former, or never drinker) and physical exercise.

**Table S6 Results from the WQS regression analysis for examining the link between metal mixtures and LDL-C**

| Indicator | Positive index | |  | Negative index | |
| --- | --- | --- | --- | --- | --- |
|  | Estimate | *P* |  | Estimate | *P* |
| intercept | 4.330 | 0.000 |  | 5.210 | 0.000 |
| wqs | 0.326 | 0.058 |  | -0.245 | 0.093 |
| Smoking | -0.330 | 0.105 |  | -0.369 | 0.069 |
| Drinking | 0.116 | 0.663 |  | 0.146 | 0.582 |
| Physical activity | 0.105 | 0.766 |  | 0.048 | 0.893 |
| BMI | -0.031 | 0.191 |  | -0.027 | 0.250 |

Abbreviations: Weights of WQS regression models were assigned to each metal in positive direction and negative direction; LDL-C, low-density lipoprotein cholesterol; Model was adjusted for body mass index (BMI), smoking status (current, former, or never smoker), alcohol consumption (current, former, or never drinker) and physical exercise.


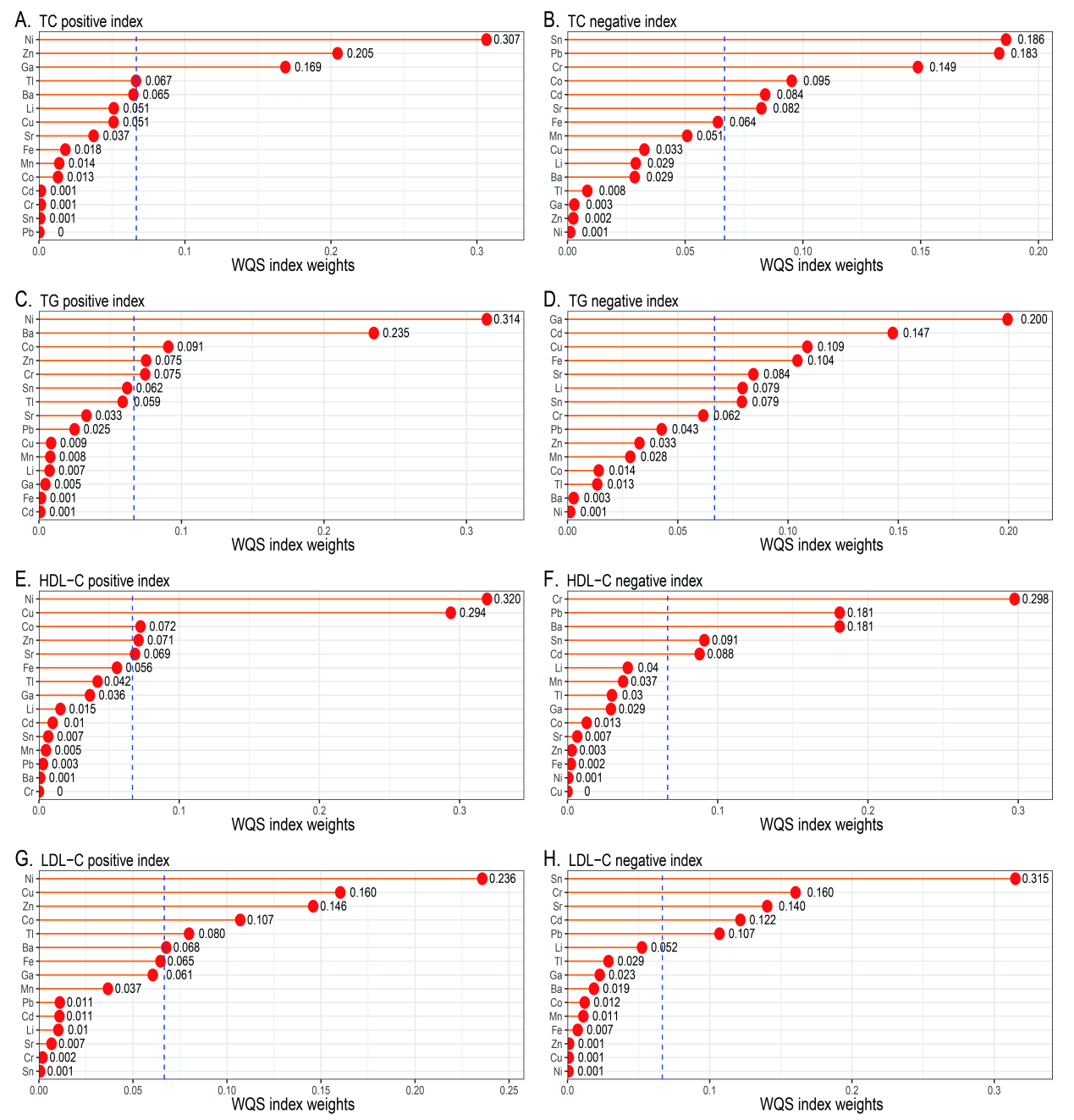


**Fig. S4** **WQS model regression index weights for TC, TG, HDL-C and LDL-C. (A)** WQS index weights of the positive effect of elements on TG. **(B)** WQS index weights of the negative effect of elements on TG. **(C)** WQS index weights of the positive effect of elements on TC. **(D)** WQS index weights of the negative effect of elements on TC. **(E)** WQS index weights of the positive effect of elements on HDL-C. **(F)** WQS index weights of the negative effect of elements on HDL-C. **(G)** WQS index weights of the positive effect of elements on LDL-C. **(H)** WQS index weights of the negative effect of elements on LDL-C. The blue dotted line represents a weight that exceeds the reference standard (where the weight is more than 1/15 of the reference standard). Model was adjusted for body mass index (BMI), smoking status (current, former, or never smoker), alcohol consumption (current, former, or never drinker) and physical exercise.

**Table S7 Collinearity Statistics**

| Variables | Tolerance | VIF |
| --- | --- | --- |
| 7Li | 0.683 | 1.464 |
| 52Cr | 0.319 | 3.133 |
| 55Mn | 0.061 | 16.338 |
| 56Fe | 0.109 | 9.182 |
| 59Co | 0.956 | 1.046 |
| 60Ni | 0.069 | 14.435 |
| 64Cu | 0.403 | 2.482 |
| 65Zn | 0.746 | 1.34 |
| 69Ga | 0.631 | 1.586 |
| 87Sr | 0.544 | 1.839 |
| 112Cd | 0.617 | 1.621 |
| 118Sn | 0.91 | 1.099 |
| 137Ba | 0.923 | 1.083 |
| 204Tl | 0.729 | 1.372 |
| 207Pb | 0.709 | 1.410 |


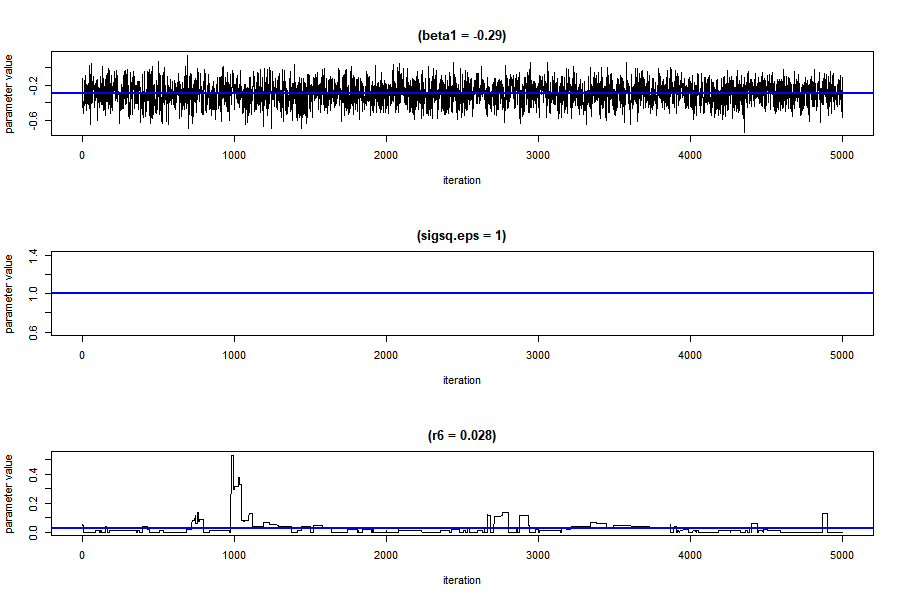


**Fig. S5 Validation of the convergence of the BKMR model**. Display iterate 5000 times (default display after 50%)

**Table S8 Interactions between metal elements in conditional logistics regression**

| Term of interaction | *β* | SE | Z | *p* |
| --- | --- | --- | --- | --- |
| log10(Li) : log10(Mn) | 1.059 | 0.664 | 1.595 | 0.110 |
| log10(Li) : log10(Sn) | -0.416 | 0.264 | -1.574 | 0.120 |
| log10(Li) : log10(Ni) | -1.004 | 0.466 | -2.154 | 0.031 |
| log10(Sn) : log10(Mn) | -0.475 | 0.197 | -2.412 | 0.016 |
| log10(Ni) : log10(Mn) | 0.628 | 0.188 | 3.336 | <0.001 |
| log10(Ni) : log10(Sn) | 0.091 | 0.435 | 0.210 | 0.830 |

**Table S9** The sensitivity analysis for the association between metal element and abnormal lipid metabolism.

| Elements concentration | *OR* (95%*CI*) | | |
| --- | --- | --- | --- |
|  | Crude model^a^ | Adjusted model^b^ | Multi-element model^c^ |
| 7Li | 0.749 (0.537, 1.169) | 0.768(0.567, 1.048) | **0.667 (0.438, 0.879)** |
| 52Cr | **0.643 (0.480, 0.898)*** | **0.627 (0.445, 0.837)*** | 0.740 (0.453, 1.316) |
| 55Mn | **1.226 (1.087, 1.563)*** | 1.278 (1.021, 1.562) | 1.444 (0.506, 3.548) |
| 56Fe | 1.184 (1.044, 1.898) | 1.214 (0.970, 1.465) | 1.079 (0.688, 2.341) |
| 59Co | 1.045 (0.819, 1.168) | 1.042 (0.960, 1.360) | 1.046 (0.780, 1.162) |
| 60Ni | **1.146 (1.045, 1.371)*** | 1.147 (1.028, 1.337) | 0.830 (0.567, 1.524) |
| 64Cu | 1.974 (0.877, 4.565) | 1.840 (0.763, 4.059) | 2.750 (0.966, 7.159) |
| 65Zn | **0.321, (0.143, 0.785)*** | **0.302 (0.132, 0.638)*** | 0.476 (0.182, 1.228) |
| 69Ga | 1.023 (0.970, 1.164) | 1.120 (0.932, 1.139) | 1.143 (0.866, 1.197) |
| 87Sr | 0.924 (0.568, 1.768) | 0.875 (0.441, 1.675) | 0.616 (0.223, 1.431) |
| 112Cd | 0.845 (0.735, 1.024) | 0.844 (0.768, 1.032) | 0.995(0.825, 1.145) |
| 118Sn | **0.775 (0.640, 0.920)*** | **0.744 (0.645, 0.864)*** | **0.812 (0.649, 0.985)** |
| 137Ba | 1.045 (0.812, 1.484) | 1.165 (0.825, 1.764) | 1.275 (0.945, 1.842) |
| 204Tl | 1.030 (0.944, 1.245) | 1.144 (0.950, 1.475) | 1.144 (0.966, 1.342) |
| 207Pb | 0.858 (0.865, 1.046) | 0.914(0.792, 1.045) | 0.785 (0.645, 1.214) |

Abbreviations: Li, Lithium-7; 52Cr, Chromium-52; 55Mn, Manganese-55; 56Fe, Iron-56; 59Co, Cobalt-59; 60Ni, Nickel-60; 64Cu, Copper-64; 65Zn, Zinc-65; 69Ga, Gallium-69; 87Sr, Strontium-87; 112Cd, Cadmium-112; 118Sn, Tin-118; 137Ba, Barium-137; 204Tl, Thallium-204; 207Pb, Lead-207; ^a^ Crude model was single element expressed by crude odds ratio (95% confidence interval); ^b^ Adjusted model was single element adjusted for body mass index (BMI), smoking status (current, former, or never smoker), alcohol consumption (current, former, or never drinker) and physical exercise; ^c^ Multi-element model included fifteen serum metal elements into analysis and was adjusted for body mass index (BMI), smoking status (current, former, or never smoker), alcohol consumption (current, former, or never drinker), physical exercise (yes or no), drinking tea (yes or no) and education levels (no formal education, primary school, middle school or beyond); *, After false discovery rate (FDR) correction (q < 0.05).
